# RNA sequencing from neural ensembles activated during fear conditioning in the mouse temporal association cortex

**DOI:** 10.1038/srep31753

**Published:** 2016-08-25

**Authors:** Jin-Hyung Cho, Ben S. Huang, Jesse M. Gray

**Affiliations:** 1 Harvard Medical School, Genetics Department, 77 Ave Louis Pasteur NRB Room 356, Boston, Massachusetts 02115, USA; 2 University of California at Los Angeles, David Geffen School of Medicine, Department of Neurology, 710 Westwood Plaza, Los Angeles, California 90095, USA.

## Abstract

The stable formation of remote fear memories is thought to require neuronal gene induction in cortical ensembles that are activated during learning. However, the set of genes expressed specifically in these activated ensembles is not known; knowledge of such transcriptional profiles may offer insights into the molecular program underlying stable memory formation. Here we use RNA-Seq to identify genes whose expression is enriched in activated cortical ensembles labeled during associative fear learning. We first establish that mouse temporal association cortex (TeA) is required for remote recall of auditory fear memories. We then perform RNA-Seq in TeA neurons that are labeled by the activity reporter *Arc-dVenus* during learning. We identify 944 genes with enriched expression in *Arc*-*dVenus*+ neurons. These genes include markers of L2/3, L5b, and L6 excitatory neurons but not glial or inhibitory markers, confirming *Arc-dVenus* to be an excitatory neuron-specific but non-layer-specific activity reporter. Cross comparisons to other transcriptional profiles show that 125 of the enriched genes are also activity-regulated *in vitro* or induced by visual stimulus in the visual cortex, suggesting that they may be induced generally in the cortex in an experience-dependent fashion. Prominent among the enriched genes are those encoding potassium channels that down-regulate neuronal activity, suggesting the possibility that part of the molecular program induced by fear conditioning may initiate homeostatic plasticity.

The recall of remote fear memories–those that persist for days, weeks, or longer–requires the cerebral cortex in addition to the amygdala and other regions^1^,^2^. For cued fear memories, the sensory cortex in particular is an appealing region where a remote association between a conditioned sensory cue and an unconditioned stimulus may be formed, producing a memory engram^3^. Consistent with this idea, sensory cortex is required for recall of remote but not recent associations^4^, and the activity reporter *Egr1* is induced in sensory cortex upon remote but not recent recall^5^. However, *Egr1* is just one member of a larger experience-dependent gene program (including *Fos*, *Npas4*, *Arc*) whose components are induced during learning and thought to be required for long-term memory formation^6^,^7^,^8^,^9^.

Until recently, limitations in genomic technologies have precluded identification of the most functionally relevant set of fear conditioning-induced genes in the cortex. The cortex contains diverse cell types, and the activity-regulated gene programs differ among them^10^. Due to this cell-type diversity, detecting gene expression changes in bulk tissue inevitably entails a loss in sensitivity. Although RNA Sequencing (RNA-Seq) is an improvement over microarray methods, technical issues including batch effects and read depth still limit sensitivity^11^. This limited sensitivity poses a serious challenge for the identification of the neural activity-induced “effector” genes that alter circuit function, as these genes are expressed at lower levels than immediate-early transcription factors^7^,^12^. RNA-Seq has now been combined with cell-type specific labeling to profile experience-dependent gene expression^13^,^14^, but even these approaches measure gene expression in large populations of cells, only a subset of which is responsive to experience. With the goal of identifying potential effector genes, we performed RNA-Seq specifically in neural ensembles in sensory association cortex that are engaged during fear conditioning.

## Results

**Figure 1:**
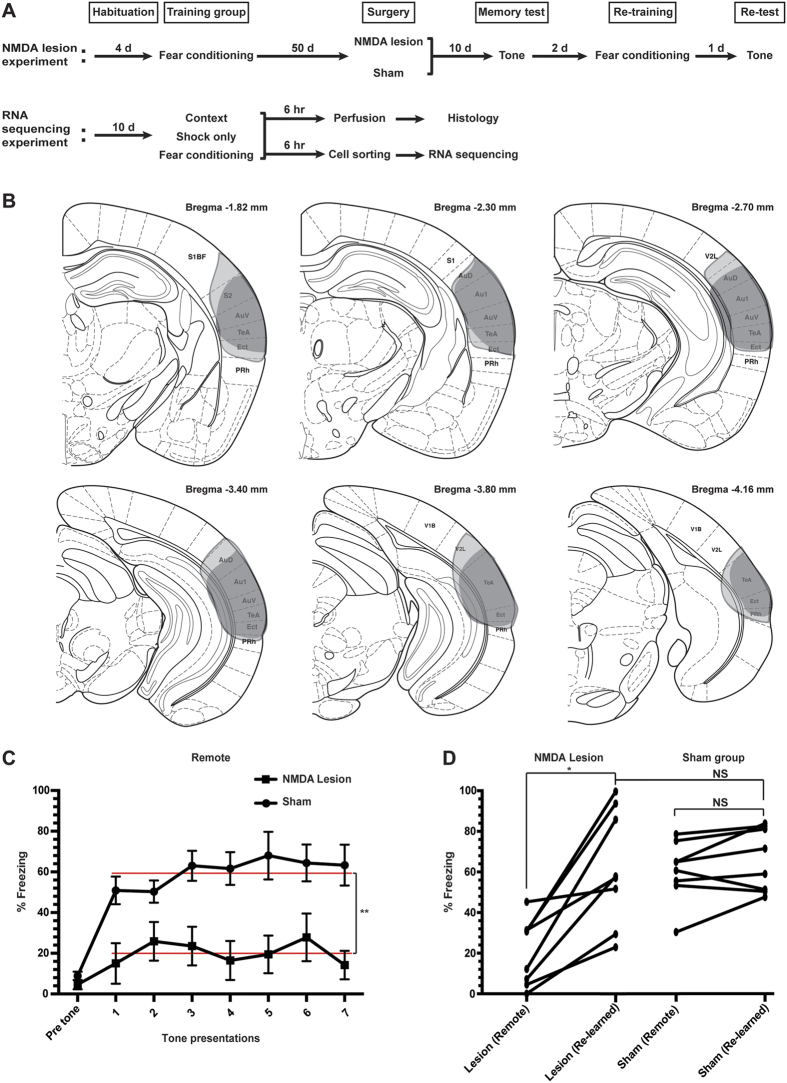
TeA is required for remote fear memory recall in mice. (**A**) Experimental design for behavioral testing and RNA sequencing. (**B**) The extent of lesioning in the two mice with the smallest (dark grey) and largest (gray) lesions of the group. Coronal brain section images adapted from^42^. S1BF, primary somatosensory cortex barrel; S2, secondary somatosensory cortex; V2L, secondary visual cortex lateral area; AuD, secondary auditory cortex dorsal; AuV, secondary auditory cortex ventral; TeA, temporal association cortex; Ect, ectorhinal cortex; PRh, perirhinal cortex. (**C**) Remote auditory fear memory in auditory cortex lesioned (n = 8) and sham-operated (n = 8) animals. Fear response was measured as percentage of total freezing both 7 min before (pre tone) and during (test trial) presentation of seven CSs. All values are means +/− SEM. Red lines represent mean freezing across the indicated presentations. **p = 0.0002, unpaired *t*-test (two-tailed). (**D**) Auditory cortex lesioned animals were able to reacquire CS-US association. Each data point represents the mean freezing across all seven CS presentations for each animal. *p = 0.0022, Paired *t*-test (two-tailed); NS, not significant.

Our experimental design is outlined in Fig. 1A. In auditory fear conditioning experiments, we habituated mice to handling and the conditioning chamber for 4 or 10 days, without tones (the conditioned stimuli) or shocks (the unconditioned stimuli). We then performed fear conditioning with a series of seven tones and co-terminating footshocks (FC group). In control experiments, we also exposed mice to footshocks without tones (shock only group) or the chamber context only (control group). To confirm that remote memories were acquired, we assessed remote memory recall by re-presenting the tones in a new context after two months (performed in a parallel cohort). In seeking to identify gene expression relevant to remote memory consolidation, we focused our gene expression analysis on the period immediately following learning. This choice may seem counter-intuitive, given that the cortex is not required for recent auditory fear recall^4^. However, in several cortical areas, gene induction during learning occurs in the same neurons later reactivated during recall^15^. Given that inducible transcripts likely persist for hours to days, it is the gene induction during learning itself that we hypothesized is the gene induction most relevant to memory consolidation.

For the RNA-Seq experiment, we sought to target a specific area of sensory cortex that is required for remote recall of cued fear memories in mice. We chose mice for these experiments to take advantage of the high quality mouse genome annotations and associated genomic data available^12^,^13^,^16^. We focused on temporal association cortex (area TeA) because it processes auditory information and is rich in projections to the amygdala^17^,^18^. To determine whether mouse TeA is required for fear recall, we lesioned TeA after fear conditioning but before testing recall. Our lesions completely covered TeA, extending into ectorhinal and parts of auditory cortex (Au1, AuD, AuV; Fig. 1B). We found that TeA lesions dramatically attenuated remote memory recall, reducing freezing in response to tone presentation two months after training (Fig. 1C). The effect of the lesion cannot be attributed to a defect in hearing, because lesioning did not affect the ability of mice to subsequently re-associate tone and shock (Fig. 1D). These results are in agreement with those obtained from rats^4^,^19^,^20^, consistent with the idea that plasticity in TeA or nearby regions during learning or consolidation may be a substrate for remote associative memory formation.

**Figure 2:**
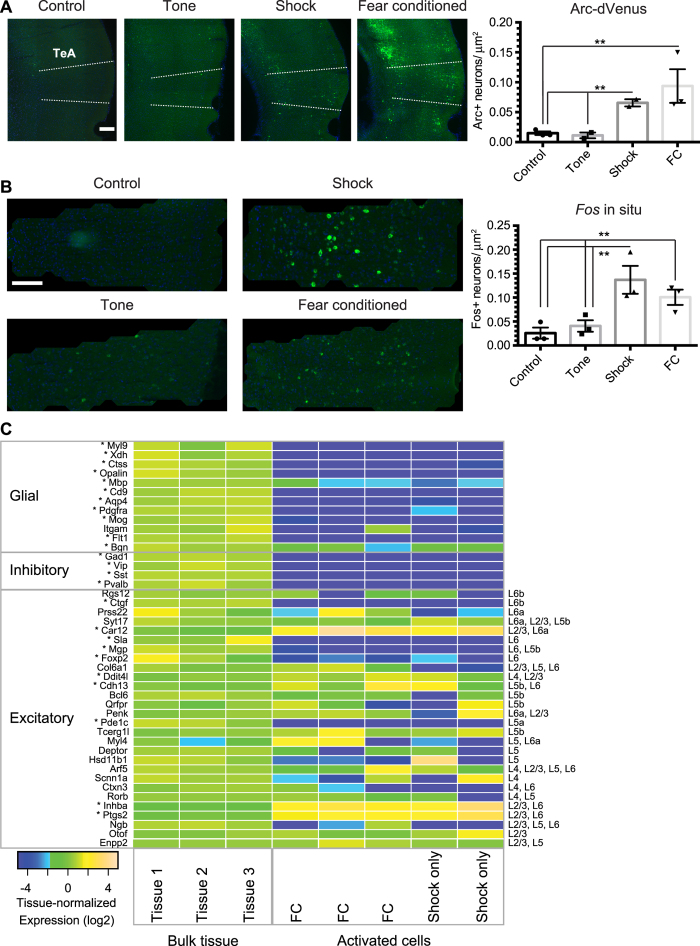
*Arc-dVenus*+ neurons are enriched for expression of L2/3, L5b, and L6a markers. (**A**) Representative images and quantification of Arc-dVenus expression in TeA from control, tone-only, shock-only, and fear-conditioned mice. Scale bar, 200 μm. All values are mean +/− SEM. **p < 0.05, unpaired *t*-test (two-tailed). Unstarred comparisons are not significantly different. N = 3 mice for control and fear conditioned groups and two mice for the tone-only and shock-only groups. (**B**) *Fos in situ* hybridization. Representative cropped images and quantification of *Fos* expression in TeA from control, tone-only, shock-only, and fear-conditioned mice. N = 3 mice for all groups. Scale bar, 200 μm. All values are means +/− SEM. **p < 0.05, unpaired *t*-test (two-tailed). (**C**) Heatmap of expression differences between bulk TeA tissue and sorted Arc-dVenus cells. Glial, inhibitory, and excitatory markers^26^,^46^ are indicated at left. Each gene’s expression is normalized to its mean across the three bulk-tissue samples. Within excitatory markers, genes are in rough order of specificity for layer 6 (top) to layer 2 (bottom). Markers for non-neural cells include *Aqp4* (astrocytes); *Pdgfra* (oligodendrocyte precursor cells, OPCs); *Mog*/*Opalin* (oligodendrocytes); *Ctss*/*Itgam* (microglia); *Flt1*/*Xdh* (endothelial cells); and *Bgn*/*Myl9* (smooth muscle cells, SMC). Bulk tissue samples are from FC (Tissue 1), shock (Tissue 2–3) mice respectively. Sorted cells are from FC (23 cells), FC (39 cells), FC (40 cells), shock (30 cells), and shock (33 cells) respectively. *Indicates false discovery rate (FDR) <0.05 from an overdispersed Poisson model.

We focused our RNA-Seq efforts on neural ensembles that are activated during fear conditioning through the use of *Arc-dVenus* activity reporter mice^21^. In these mice, the activity-regulated *Arc* promoter drives expression of a destabilized *Venus* fluorophore, labeling neurons activated within the previous 3–12 hour period. To characterize this reporter, we examined native *Arc-dVenus* fluorescence in sections from mice sacrificed six hours after fear conditioning. We reasoned that performing RNA-Seq at this timepoint would maximize our ability to detect the activity-regulated effector genes, whose induction is typically slower than that of immediate-early transcription factors. We found that *Arc-dVenus* is strongly induced in TeA six hours after fear conditioning, primarily in layer 2/3 (L2/3), layer 5 (L5), and layer 6 (L6) (Fig. 2A). The layer-to-layer pattern we observe here is similar to endogenous *Arc* mRNA expression^22^, giving us an additional measure of confidence in this established activity reporter^21^,^23^.

To investigate the potential relationship between *Arc-dVenus*+ neurons and an associative memory trace, we asked which specific elements of associative learning lead to *Arc-dVenus* induction in TeA: tone (CS), shock (US), or coincident tone-shock association. We found that expression of *Arc-dVenus* is induced by shock alone but not tone alone (Fig. 2A). Moreover, *Arc-dVenus* expression is indistinguishable in shock-only and fear conditioning conditions, suggesting that it is dependent on US rather than coincident CS/US detection. To address whether this US-dependence is specific to this particular reporter or to the *Arc* gene, we examined the mRNA expression of another well-studied immediate early gene, *Fos*, and found it also to be US- but not CS-dependent (Fig. 2B). Despite the tone having no apparent influence on *Arc-dVenus* expression, these results are consistent with the idea that an associative memory trace could be contained within a subset of *Arc-dVenus* neurons that coincidently detect tone and shock. Whether the specific *Arc-dVenus*+ neural ensemble activated in TeA during fear conditioning is reactivated during recall has not been established, although ensemble reactivation has been observed in other cortical areas^15^.

To obtain RNA from *Arc-dVenus*+ neurons, we performed cell dissociation and manual sorting^24^, isolating between 23 and 39 *Arc-dVenus*+, TeA cells per animal. The manual sorting approach produces results similar to Fluorescent-Activated Cell Sorting but is advantageous when labeled cells are sparse or tissue amount is limiting^25^. From the RNA isolated from sorted cells, we made five sequencing libraries: three from fear conditioned mice and two from shock-only control mice. We did not sequence *Arc-dVenus*+ cells from context-only controls because these mice have very few activated *Arc-dVenus*+ cells (Fig. 2A). To provide a reference point for calculating the enrichment of gene expression in *Arc-dVenus*+ cells, we sequenced three TeA bulk-tissue samples. To maximize their value as reference points, we processed these bulk-tissue samples in exactly the same way as the tissue sections that we used for cell sorting (*i.e.,* both were protease-treated identically). Two were from shock-only mice, and one was from a fear-conditioned mouse. We used both conditions for the purpose of comparing bulk-tissue to sorted *Arc-dVenus*+ neurons. In support of this approach, bulk-tissue samples were highly similar to each other and distinct from *Arc-dVenus*+ sorted cells, regardless of condition (Supplementary Fig. S1). However, our study was not designed or powered to address gene expression differences between bulk-tissue conditions. Instead, by comparing mRNAs enrichment in *Arc-dVenus*+ cells compared to bulk-tissue samples, we aimed to identify those genes that are enriched in ensembles of neurons activated during learning.

Analysis of *Arc-dVenus*+ ensembles enables detection of gene expression only in the specific cell types in which *Arc-dVenus* is expressed. To identify cell types that express the *Arc-dVenus* reporter after fear conditioning, we compared RNA-Seq data for *Arc-dVenus*+ cells and bulk-tissue controls. We found that non-neuronal markers are de-enriched in *Arc-dVenus*+ cells, including greater than 100-fold de-enrichments of the astrocyte marker *Gfap*, the microglial marker *Ctss*, and the oligodendrocyte precursor marker *Pdgfra* (*p* < 10^−5^, Fig. 2C). Markers for GABAergic inhibitory neurons are also de-enriched, including the pan-inhibitory marker *Gad1* and markers for three major classes of inhibitory neurons, *Vip*, *Sst*, and *Pvalb* (each >100-fold de-enriched, *p* < 10^−13^). In contrast, the pan-excitatory marker *Slc17a7* (Vglut1) is enriched 11-fold (*p* < 10^−6^). Thus, analysis of the *Arc-dVenus*+ transcriptome does not permit detection of inhibitory neuron-specific gene expression but does enable detection of excitatory neuron-specific gene expression. Since principal neurons in the cortex are excitatory, Arc-dVenus reporter mice are well-suited for identifying genes enriched in activated principal neurons.

To identify subtypes of excitatory neurons that express *Arc-dVenus*, we examined the *Arc-dVenus* enrichment of 28 markers that distinguish subtypes of cortical excitatory neurons^26^ (Fig. 2C). The layer 2/3 (L2/3) markers *Ptgs2* and *Inhba* are greater than 300-fold enriched (*p* < *10*^−16^). However, the L4-specific *Scnn1a* and *Arf5* are not significantly enriched (<1.5-fold, *p* > 0.93). The L5b markers *Cdh13, Tcerg1l*, and *Qrfpr* are expressed in *Arc-dVenus*+ neurons and trend toward positive enrichment (33-fold, 14-fold, 1.8-fold; *p* = *0.002, 0.06,* 0.86). The L6a markers *Car12* and *Myl4* also have positive enrichment (>9-fold, *p* = 0.02 and 0.52). The prominence of L2/3, L5, and L6 markers is consistent with the abundance of *Arc-dVenus* fluorescence in these layers (Fig. 2A) and suggests that our approach is well-powered to detect fear conditioned-induced genes in the excitatory neurons in these layers.

We asked whether global gene expression in activated neurons differs following fear conditioning versus shock presentation alone. We found the *Arc-dVenus*-sorted expression profiles from fear conditioning and shock to be indistinguishable (Supplementary Fig. S2A,B). One possible explanation would be that most of the genes enriched in *Arc-dVenus*+ neurons are cell-type markers rather than neuronal activity-regulated genes. However, we found that the expression of a smaller set of *bona fide* activity-regulated genes is not differentially expressed in *Arc-dVenus*+ neurons between fear conditioning and shock conditions (Supplementary Fig. S2C). Our experiments are not sufficiently powered to rule out the possibility of potential gene expression differences between fear conditioning and shock. However, a comparison to KCl-induced gene expression in primary neurons^12^ suggests that our experiments here are adequately powered to detect a systematic difference in expression of activity-regulated genes between FC and shock conditions (Supplementary Fig. S2D,E). The lack of such systematic bias suggests that global TeA gene induction during auditory fear conditioning may not require coincident detection of a tone and shock. This idea is consistent both with similar results in the auditory cortex^27^ and with the idea that disinhibition of L2/3 neurons in sensory cortex is shock-dependent but not auditory cue-dependent^28^. In light of the indistinguishability of gene expression from shock and fear conditioning conditions, we subsequently combined data from these conditions to identify with maximal statistical power the genes whose expression is elevated in *Arc-dVenus*+ neurons compared to bulk-tissue. Our decision to pool all sorted samples together is supported by our analysis showing that gene expression differs much more between sorted neurons and bulk-tissue than between sorted conditions (Supplementary Fig. S1).

**Figure 3:**
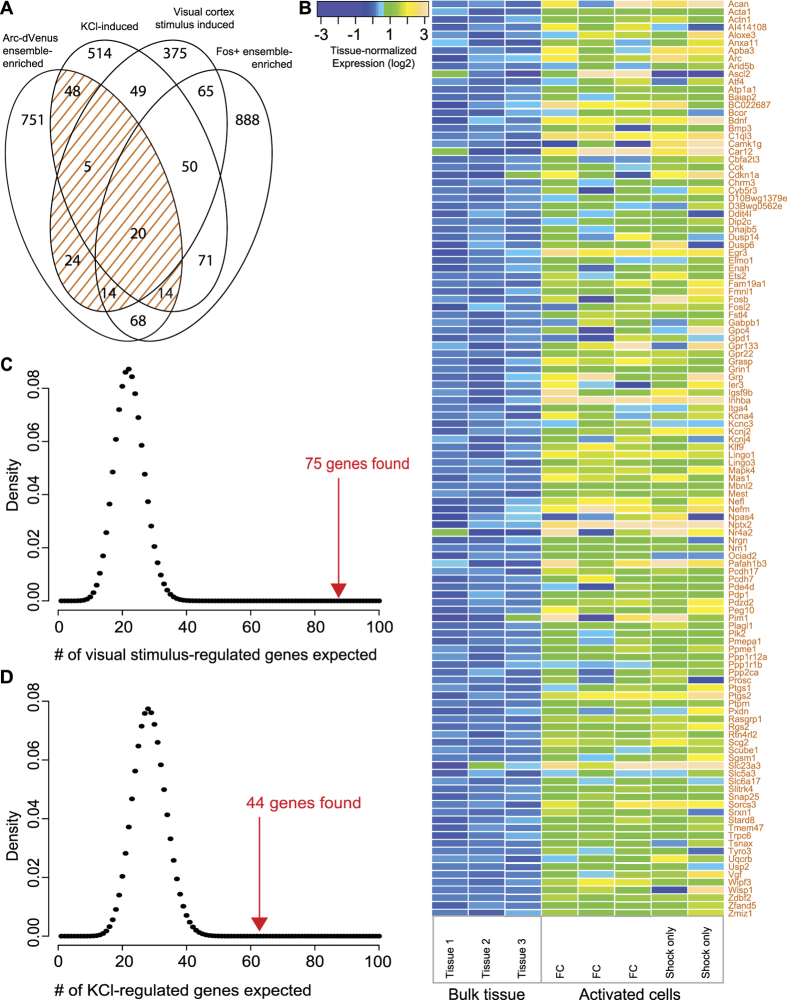
Activity-regulated genes enriched in Arc-dVenus+ neurons. (**A**) Venn diagram showing overlap between *Arc-dVenus*-enriched genes, genes regulated by activity *in vitro*^12^, visual stimulus-regulated genes^13^, and genes enriched in *Fos*+ hippocampal neurons^29^. *Arc-dVenus*-enriched genes are defined by FDR <0.05 and fold-enrichment of >2 in *Arc-dVenus*+ neurons (n = 5) compared to bulk-tissue controls (n = 3). (**B**) Heatmap of expression in *Arc-dVenus*+ neurons and bulk-tissue for genes that are both Arc-dVenus-enriched and activity- or visual stimulus-regulated. Each gene’s expression is normalized to its mean across the three bulk-tissue samples. (**C**,**D**) The expected versus actual numbers of *Arc-dVenus*-enriched genes that are also visual stimulus-induced (**C**) or activity-induced (**D**) (*p* < 10^−20^, hypergeometric tests). Distributions of expected numbers from the hypergeometric distribution are in black; actual numbers are in red.

We identified 944 *Arc-dVenus*-enriched genes, which have >2-fold enrichment in *Arc-dVenus*+ neurons (over bulk-tissue) and a false discovery rate of less than 5%. To identify the subset of these genes likely to be induced by fear conditioning, we compared them to genes previously shown to be either activity-regulated *in vitro*^12^ or induced upon first exposure to visual stimulus in the visual cortex^13^. We found 125 activity-regulated *Arc-dVenus*-enriched genes, which are *Arc-dVenus*-enriched and regulated by neuronal activity or visual stimulus (Fig. 3A,B, Supplementary Table S1). The overlap of *Arc-dVenus*-enriched genes with activity-regulated and visual stimulus-induced genes is highly significant (each *p* < 10^−20^, hypergeometric test) (Fig. 3C,D). In addition, most activity regulated genes, including the generally cell type non-specific early genes *Fos*, *Fosb*, and *Junb*, have higher expression in *Arc-dVenus* neurons (*p* = 2^*^10^−5^ from permutation, Supplementary Fig. 3 and Supplementary Table 1). Moreover, 116 *Arc-dVenus*-enriched genes are also enriched in *Fos*+ neurons in the hippocampus^29^ (*p* = 10^−23^, hypergeometric test). These results do not provide direct evidence that genes enriched in *Arc-dVenus*+ neurons are induced during fear learning or even that *Arc-dVenus*+ cells exhibit more learning-associated gene induction than *Arc-dVenus-* cells. However, the highly significant overlap of *Arc-dVenus*-enriched with experience-regulated genes suggests that this overlapping set of genes may be induced during fear learning in *Arc-dVenus*+ neurons.

The overlap of *Arc-dVenus*-enriched genes with visual stimulus-induced genes suggests that the plasticity that occurs during remote memory formation could be related to visual stimulus-dependent homeostatic plasticity^30^. We therefore asked whether activity-regulated *Arc-dVenus*-enriched genes are enriched for particular functions. Analysis for gene category enrichment revealed enrichment for genes whose products target the plasma membrane (*p* < 0.05 with Benjamini correction). Interestingly, activity-regulated *Arc-dVenus*-enriched genes are significantly enriched for potassium channels (*Kcna4*, *Kcnc3*, *Kcnj2*, and *Kcnj4*, p < 0.0002, hypergeometric test). Reinforcing this finding, several additional activity-regulated potassium channel genes trend toward enrichment in *Arc-dVenus*+ neurons but narrowly miss our FDR or fold-change thresholds (*Hcn1*, *Kcnj3*, *Kcnh7*; all 2-fold enriched with FDR <10%). Moreover, three of the activity-regulated, *Arc-dVenus*-enriched potassium channels (*Kcnc3*, *Kcnj2*, *Kcnj4*) are also enriched in *Fos*+ neurons in the hippocampus^29^. The enrichment of potassium channel transcripts among activity-regulated *Arc-dVenus*-enriched genes suggests that fear conditioning may lead to homeostatic decreases in the excitability of activated neural ensembles.

## Discussion

In this study, we set out to profile gene expression in neural ensembles that are activated during fear learning. We used the well-tested auditory fear conditioning paradigm in mice. We first established that the temporal association cortex (TeA) is necessary for recall of remote auditory fear memories. Next, using *Arc-dVenus* reporter mice, we sequenced RNA specifically from neural ensembles in TeA that are activated during fear conditioning. These ensembles are composed of excitatory neurons, as they are both enriched for layer-specific excitatory neuronal markers as well as de-enriched for inhibitory neuronal and glial markers. We found that the genes expressed in these ensembles overlap with those induced *in vitro* by depolarization and in the visual cortex by visual stimuli, suggesting that these genes may contribute to activity-dependent plasticity. Among these genes are multiple potassium channel-encoding transcripts, suggesting that fear conditioning may initiate a homeostatic response that dampens the activity of the cortical ensembles that it most strongly activates.

Cortical neurons are known to exhibit non synapse-specific, homeostatic decreases in activity that can be transcription-dependent. These include not only *Arc*-dependent plasticity of synaptic strength^31^,^32^, but also plasticity of intrinsic excitability^33^, which is dynamically regulated in visual cortex pyramidal neurons via regulation of potassium conductance^34^. Furthermore, the specific neural ensembles defined by *Arc*+ or *Fos*+ expression have lower intrinsic excitability^35^,^36^ and fewer spines^37^ than their neighbors, consistent with the idea that they may be undergoing homeostatic downregulation. Although such downregulation could function exclusively to preserve homeostasis, plasticity of intrinsic excitability is also a fundamental mechanism of encoding information in circuits^38^. One possibility is that decreased intrinsic excitability could desensitize the activity of footshock-responsive cortical ensembles to subsequent footshock presentations, thereby protecting against chronic pain or stress. In contrast to these ideas, the activity-regulated gene program has also long been suggested to be required for Hebbian increases in synaptic strength^39^,^40^. Yet it has not been clear whether Hebbian plasticity, homeostatic plasticity, or both modes of plasticity are engaged in the specific neural ensembles activated during fear conditioning. Our RNA-sequencing analyses provide fresh support for the importance of transcription-dependent homeostatic responses in these activated ensembles.

## Method and Materials

### Animals

All animal studies were approved by and performed in accordance with the guidelines of the Institutional Animal Care and Use Committee (IACUC) at Harvard University. P60 to P90 male wild-type C57BL/6 mice (Charles River) were used for excitotoxic NMDA lesions, remote memory recall tests, and *Fos* Fluorescent *in situ* hybridization; For RNA sequencing. P60 to P90 Arc-dVenus transgenic male C57BL/6 mice were used^21^. All animals were housed individually for 4 days prior to all experiments. Behavioral studies were conducted between 1:30 and 2:00 pm, except for the RNA sequencing group, which was conducted between 9:00 and 10 am. All experiments were performed during the light-phase of the mice’s daily cycle.

### Behavioral procedures

#### Fear conditioning

The mice were conditioned in standard operant conditioning chamber apparatus (Med Associates) located in the NeuroBehavior Laboratory at Harvard Medical School. For the NMDA lesion study, individually housed mice were habituated to the conditioning chambers for 20 min for 4 consecutive days. For the RNA sequencing study, the mice were habituated to the conditioning chambers for 10 consecutive days prior to experiment, to ensure low background gene expression. For the NMDA lesion study (n = 16), fear conditioning (FC) consisted of presenting 7 repetitions of a CS tone (70 +/− 5 dB, 6 kHZ) terminating with the US (1-s footshock, 0.5 mA DC; inter-trial interval: 20–180 s). The animals were returned to their home cages right after the FC.

For the RNA sequencing study, the Arc-dVenus transgenic mice were divided into three different groups. One group (FC, n = 3) went through the same FC protocol as the NMDA lesion group and received 7 CS and US pairing. Another group (Shock, n = 2) received 7 repetitions of US (1-s footshock, 0.5 mA DC; inter-trial interval: 20–180 s) without any CSs. The bulk-tissue controls included two mice that were shocked without tones, and one mouse that was fear conditioned. All Arc-dVenus transgenic mice were sacrificed 6 hrs after the behavioral procedures.

For *Fos* expression analysis, the mice were conditioned in custom operant conditioning chambers described in a previous study^41^. The mice were divided into four different groups. The first group (FC, n = 3) went through the same FC protocol as the NMDA lesion group and received 7 CS and US pairings. The second group (Tone, n = 3) received 7 repetitions of CS (70 +/− 5 dB, 6 kHZ; inter-trial interval: 20–180 s) without any USs. The third group (Shock, n = 3) received 7 repetitions of US (1-s footshock, 0.5 mA DC; inter-trial interval: 20–180 s) without any CSs. The last group (Control, n = 3) was put into the conditioning chamber and did not receive any CS nor US. All mice were sacrificed 1 hr after the behavioral procedures.

#### Remote memory test

On the 60th day after FC (10 days after NMDA lesion surgery), auditory remote fear memory was assessed by placing the animals in a new environment and measuring the freezing response to 7 presentations of CS+. Freezing measurements were automated using the FreezeScan system (Clever sys.). The animals were returned to their home cages right after the recall test.

#### Retrained and recent memory test

Two days after assessing their remote memory, the animals were retrained to the same CS (70 +/− 5 dB, 6 kHZ) using the same FC protocol (above), and 1 day later, their recent memory was assessed by measuring the freezing response to the same 7 presentation of CS+. After the recall test, the mice were sacrificed to assess the size of the lesions.

### Excitotoxic NMDA lesion surgery

50 days after the FC, excitotoxic lesion of TeA was performed on 8 mice which were randomly chosen from 16 animals that were fear conditioned. Mice were anaesthetized with isoflurane (5% for induction, 1–1.5% for maintenance) in a Kopf stereotaxic apparatus. In order to lesion the entire TeA, the mice (n = 8) received two injections per hemisphere using an infusion needle (33 gauge) connected to a microsyringe pump (UMP3;WPI) and controller (Micro4;WPI) to deliver 20 μg/μl NMDA (0.3 μl/site;Sigma), dissolved in phosphate-buffered saline (PBS) at a rate of 0.1 μl/min. After the injection, the needle was left for 5 min to ensure diffusion of NMDA into the target structure and was then slowly retracted. Infusion sites were as follows (in mm): AP = −2.06 and −3.80 (from bregma), DV = −0.68 (from surface of the cortex) and ML = +/−3.75 mm^42^. The sham-operated controls (n = 4) were infused with PBS alone instead of NMDA at the same coordinates, and control mice (n = 4) only received surgery consisting of a craniotomy and no injections. These two groups were pulled into one sham group because the freezing behaviors were indistinguishable between the two (unpaired *t*-test (two-tailed), p = 0.3321). All mice that underwent surgery were allowed to recover for 10 days before being subjected to the remote recall test.

### Histology

#### Brain preparation and staining

For the excitotoxic NMDA lesion group, the animals were rapidly sacrificed with carbon dioxide after the last recall test, and their brains were rapidly removed and frozen using dry ice in methylbutane. All the brains were stored at −80 °C until cryosectioning. Coronal sections (20 μm) were cut on a cryostat and were counter-stained using hematoxylin nuclear counterstain (Vector lab) following the manufacturer’s recommendation.

To assess the expression pattern of Arc-dVenus in control (n = 3), tone (n = 2), shock (n = 2) and fear conditioned mice (n = 3), the Arc-dVenus transgenic mice were deeply anaesthetized with isoflurane 6 hrs after behavioral procedures and perfused transcardially with ice-cold PBS containing 4% paraformaldehyde. The brains were dissected, post-fixed overnight at 4 °C and cryoprotected in PBS containing 30% sucrose before freezing. All of the brains were stored at −80 °C until cryosectioning. Coronal sections (20 μm) were cut on a cryostat, mounted and imaged.

To assess the expression pattern of *Fos* in control, tone, shock and fear conditioned mice, the animals (n = 3/group) were rapidly sacrificed with carbon dioxide 1 hr after behavioral procedures and their brains were rapidly frozen using dry ice in methylbutane. All the brains were stored at −80 °C until the cryosectioning. Coronal sections (20 μm) were cut on a cryostat and stored the slides at −20 °C until further use.

Fluorescent *in situ* hybridization. Nonradioactive, digoxigenin (DIG)-labeled cRNA probes with either sense or antisense orientation were synthesized by *in vitro* transcription using DIG labeling mix (Roche) according to the recommendations of the manufacturer. Probes were synthesized from cDNA clones encoding *Fos* purchased from Dharmacon (MMM1013-202760329). For fluorescent *in situ* hybridization (FISH), all solutions were prepared using RNase-free reagents and diethylpyrocarbonate (DEPC)-treated double deionzide water (ddH_2_O).

The previously cryosectioned slides were allowed to dry for 20 min. After fixation, the sections were washed with PBS 3 times for 5 min and treated with 0.3% H_2_O_2_ (vol/vol) in PBS for 30 min, followed by 3 rounds of 5 min wash with PBS. The sections were then acetylated by 0.1% acetic anhydride (vol/vol) in triethanolamine for 10 min, followed by 2 rounds of 5 min wash with PBS and 1 round of 5 min wash with 2XSSC. Afterward, the sections were incubated in the hybridization solution (Sigma) without probe for 2 h at 70 °C and incubated in the hybridization solutions with *Fos* probe for 16–18 h at 70 °C. After hybridizations, the sections were washed in 5XSSC and 2XSSC for 10 min at 65 °C, followed by 30 min wash in 50% formamide (vol/vol) in 0.2X SSC at 65 °C. The sections were further washed in 0.2XSSC at room temperature and followed by one wash in TN buffer (0.1 M Tris-HCl, pH 7.5, 0.15 M NaCl) for 5 min. The sections were blocked with 1% blocking reagent (wt/vol) (Roche) in TN buffer for 1 h and incubated in the same solutions with digoxigenin antibody conjugated to horseradish peroxidase (1:1000) (PerkinElmer) for 1 h 30 min. Following the incubation with antibody, the sections were washed 3 times in TNT buffer (0.1 M Tris-HCl, pH 7.5, 0.15 M NaCl, 0.05% Tween-20 (vol/vol)) for 5 min and incubated in the amplification solution with Fluorescein plus tyramide (1:50) (PerkinElmer) for 30 min. After the amplification, the sections were washed 3 times in TNT buffer and incubated with Hoechst (1:10000) (Invitrogen) in PBS for 5 min. After 3 rounds of washing in PBS, the sections were mounted and stored at 4 °C until further analysis.

### Microscopy and image analysis

All hemotoxyline stained, Arc-dVenus images and *Fos* FISH images were acquired with a 10X objective lens using an Olympus VS120 Whole slide scanner, which generates a proprietary vsi image at the Harvard Neurobiology Imaging Facility (NS072030).

### Data analysis

Arc-dVenus and *Fos*+ neurons were manually counted by a blinded experimenter. TeA images were quantified from 3 sections per animal.

Statistical analyses were performed using commercial software (GraphPad Prism; GraphPad Software, Inc.) for analyzing freezing data between NMDA lesion and sham groups and quantification for Arc-dVenus and *Fos*+ neurons.

### RNA sequencing

#### Manual cell sorting and RNA sequencing

Manual sorting of fluorescent cells was carried out as first described in^24^. Briefly, Arc-dVenus transgenic male C57BL/6 mice were sacrificed under isoflurane anesthesia 6 hrs after behavioral procedures and their brains were quickly removed and transferred into ice-cold oxygenated ACSF, containing 126 mM NaCl, 20 mM NaHCO_3_, 20 mM dextrose, 3 mM KCl, 1.25 mM NaH_2_PO_4_, 2 mM CaCl2, 2 mM MgCl_2_, 50 mM APV, 20 mM DNQX, 100 nM TTX. Acute 330 μm coronal brain slices were prepared and incubated in a protease E (1.2 mg/mL) (Sigma) containing oxygenated ACSF for 50 min. After 15 min of washes in the ACSF, TeA tissue was microdissected using a pair of fine scissors under a fluorescent dissecting microscope (Leica M165FC). The dissected TeA tissue was then triturated in ACSF using a series of three Pasteur pipettes of decreasing tip diameters, and the dissociated cells were transferred into a small petri dish. With visual control under a fluorescent dissecting microscope, Venus+ neurons were aspirated into a micropipette with a 30–50 μm tip diameter and transferred into a clean petri dish. A total of 35–60 Venus+ neurons were pooled for each sample, which were immediately lysed in 50 μl of extraction buffer (PicoPure RNA isolation kit, Arcturus, Life Technologies) and total mRNA was subsequently isolated. cDNA was synthesized using Ovation RNA-Seq System V2 kit (Nugen). We obtained approximately 6 μg of cDNA from 35–60 cells from each group. Then, the cDNA library was prepared using Ovation Ultralow DRMultiplex Systems (Nugen). Sequencing was conducted on an Illumina HiSeq2000, and is available via GEO accession GSE85128.

#### Analysis of Arc-dVenus RNA-sequencing data

We aligned reads in fastq format to mouse genome mm9 using RUM version 1.11 with default settings^43^. We analyzed only uniquely aligning reads, only genes with a total of at least 20 reads per million across all samples, and only one transcript variant per gene (the highest expressed across all samples). We called differentially expressed RefSeq transcripts based on a 5% false discovery rate (FDR) and a requisite 2-fold enrichment in *Arc-dVenus*+ cells. Significance is based on p.values from edgeR using an overdispersed Poisson noise model^44^, with corresponding FDRs from the R function p.adjust. The dendrograms and heatmaps were produced by heatmap.2 in R. The set of genes analyzed and their normalized read counts in each sample are provided in Table S1. Gene set overrepresentation analysis was performed using DAVID^45^ with the RefSeq genes as a background. Potassium channel enrichment was tested with a hypergeometric test that addressed whether the number of potassium channels among activity-regulated *Arc-dVenus* genes was unexpected based in their frequency among (1) all genes whose enrichment in *Arc-dVenus*+ neurons was tested and (2) genes that are activity-regulated *in vitro* or by regulated by visual stimulus in visual cortex.

#### Analysis of published sequencing data

Markers were identified from published lists of Emx1-enriched genes^13^, S1 pyramidal neurons^46^, or layer-specific markers^26^. Activity-regulated genes *in vitro* were identified as genes whose mean normalized expression increased at least 50% in previously published RNA-Seq experiments^12^. Visual stimulus-induced genes were taken from a published list^13^.

## Additional Information

**How to cite this article**: Cho, J.-H. *et al*. RNA sequencing from neural ensembles activated during fear conditioning in the mouse temporal association cortex. *Sci. Rep.* **6**, 31753; doi: 10.1038/srep31753 (2016).

## Supplementary Material

Supplementary Information

Supplementary Information
