# Supplementary material for: RNA sequencing from neural ensembles activated during fear conditioning in the mouse temporal association cortex
Source: Sci Rep. 2016 Aug 25;6:31753. doi: 10.1038/srep31753 (PMC4997356; doi:10.1038/srep31753)
Supplement: Supplementary Information [file srep31753-s1.pdf]

Supplementary information

RNA sequencing from neural ensembles activated during fear conditioning in the mouse temporal association cortex

Jin-Hyung Cho[1], Ben S. Huang[1,2], and Jesse M. Gray[1]

[1]Harvard Medical School, Genetics Department
[2]University of California, Los Angeles, Geffen School of Medicine, Neurology Department

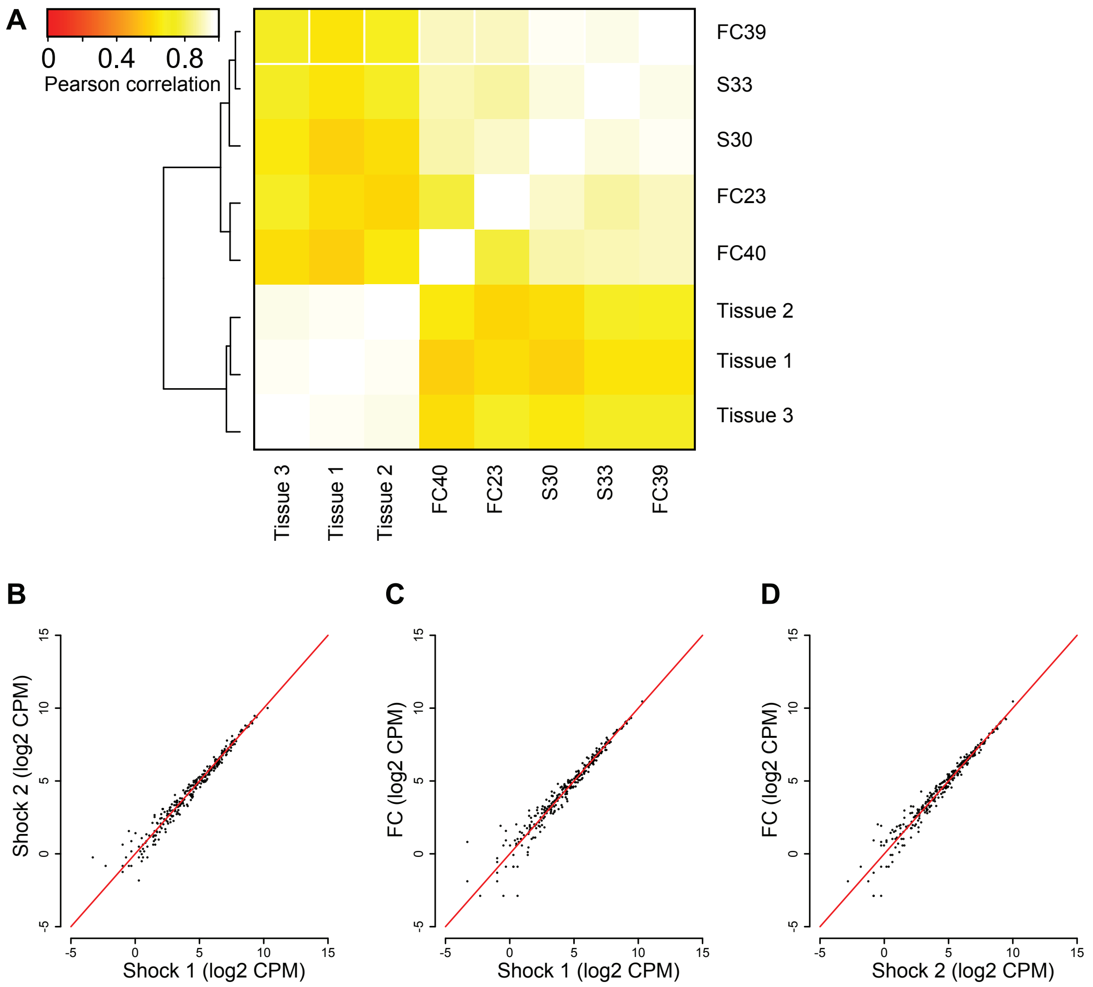

Supplementary Figure 1. Activity-regulated genes (as a group) are expressed at equivalent levels in tissue from shock and fear conditioned mice. (**A**) Pearson correlation matrix and hierarchical clustering, showing that bulk tissue samples and sorted cells cluster within their respective groups. (**B-D**) Scatterplots comparing bulk-tissue samples from three mice, where each data point is an activity- or experience-regulated gene (Methods). There are three tissue controls sequenced: shock (Tissue 2-3), and fear conditioned (FC, Tissue 1).

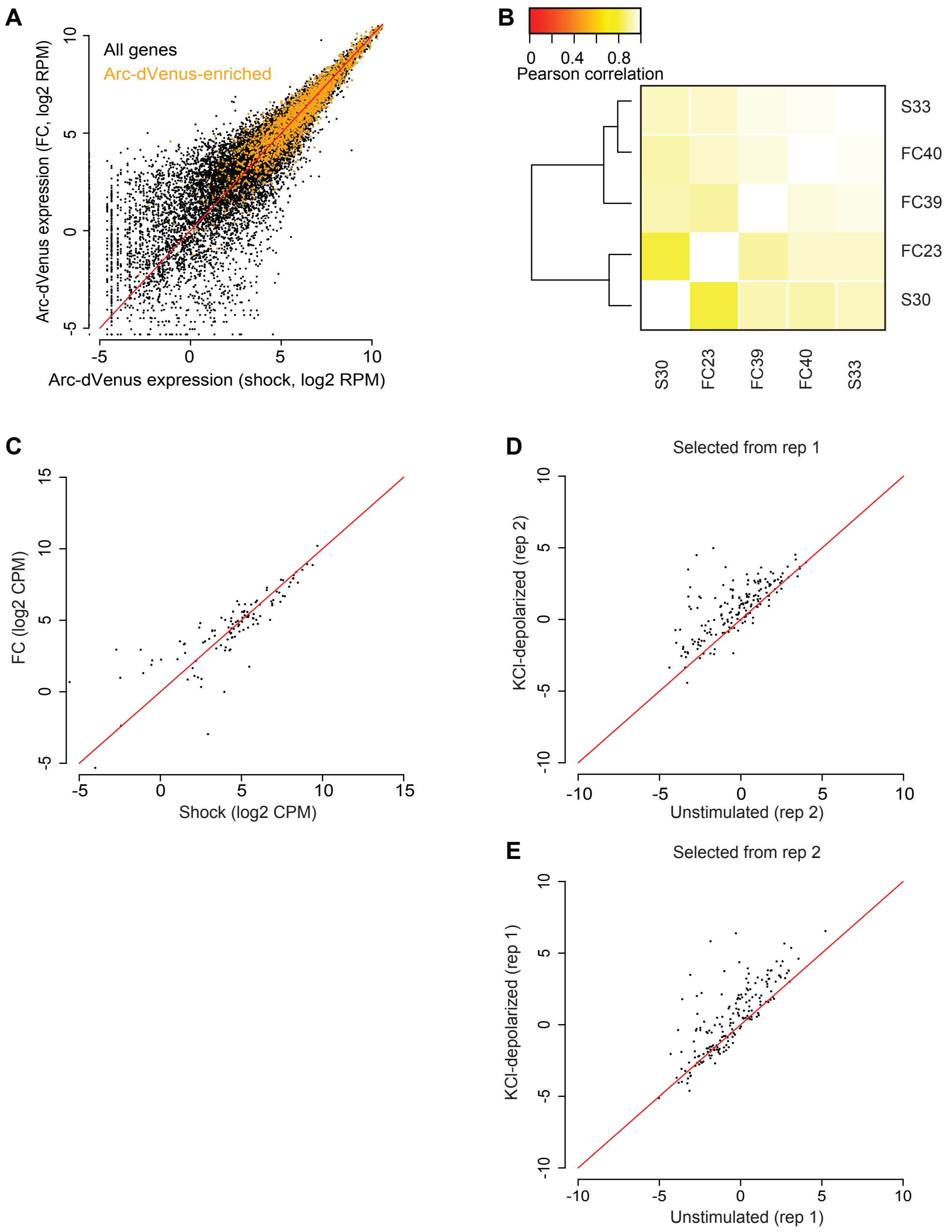

Supplementary Figure 2. Fear conditioning and shock are indistinguishable by gene expression in *Arc-dVenus*+ neurons (**A**) A scatterplot of read counts per million for fear conditioned (FC, n = 3) versus shock (n = 2) conditions (each data point is a mean across replicates). (**B**). Pearson correlation matrix and hierarchical clustering of FC and shock samples, showing that FC and shock are indistinguishable based on these data. S is shock-only, and the number that follows S or FC is the number of cells isolated and sequenced. (**C**) Activity-regulated genes do not show a bias for expression in shock or FC conditions. Each condition is based on an average across two replicates. Activity-regulated genes are from[12]. (**D-E**) To demonstrate that RNA-Seq from just one or two replicates can in principle be used to detect systematic differences between conditions, we used one replicate of a KCl-depolarization experiment [12] to identify genes strongly biased toward upregulation in a second (panel D). Panel E shows that the second replicate can similarly be used to identify genes upregulated in the first.

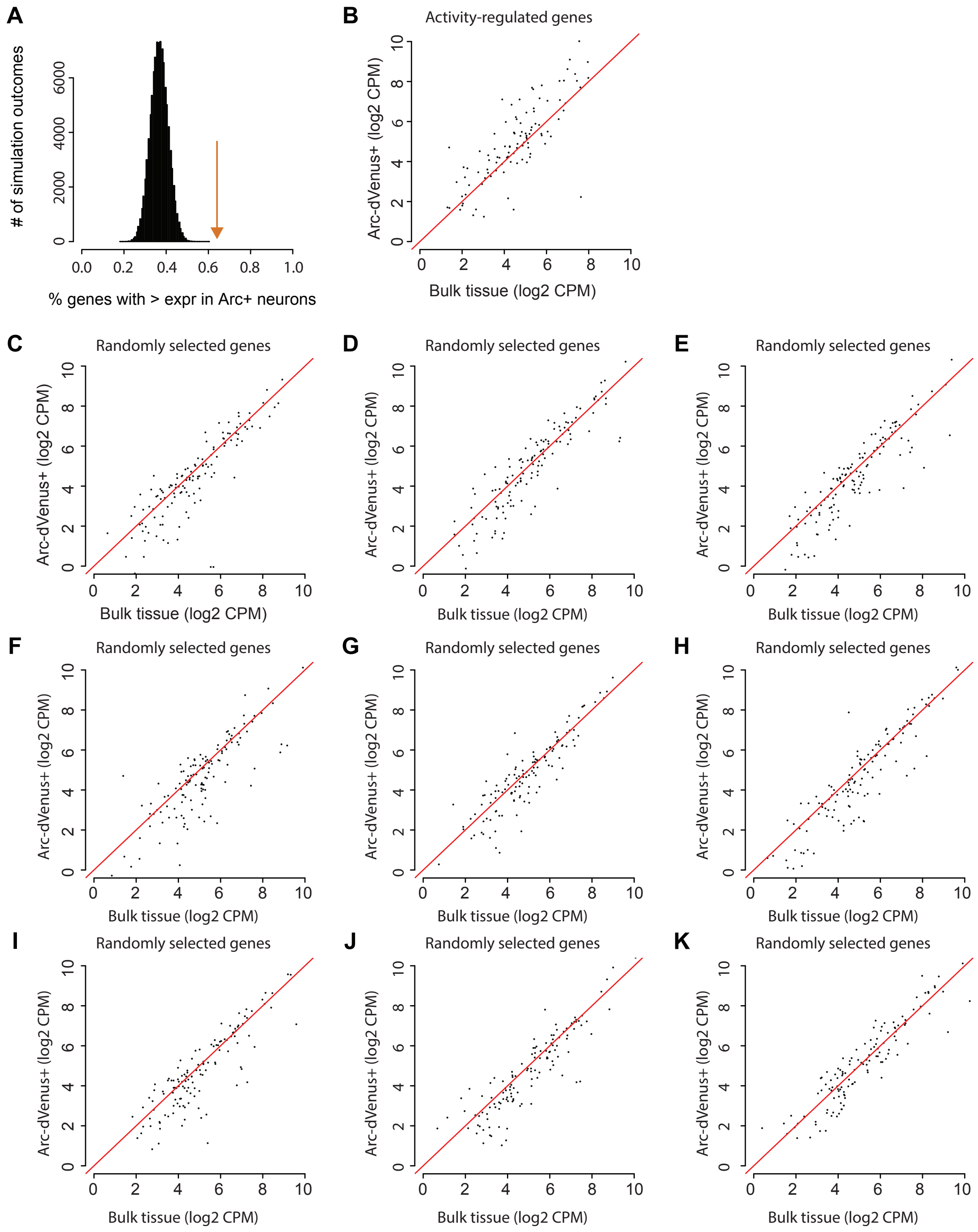

Supplementary Figure 3. (A) A histogram showing the % genes with greater expression in *Arc-dVenus*+ neurons than bulk tissue, from 100,000 simulations in which random genes were chosen. The orange arrow shows the % of activity- or experience-regulated genes with greater expression in *Arc-dVenus*+ neurons ($p = 2 * 10^{-5}$ from the simulations). **(B)** Expression of activity- or experience-regulated genes in *Arc-dVenus*+ cells versus bulk tissue. Each dot is an average from n = 5 (sorted) and n = 3 (bulk-tissue) samples. **(C-K)** *Arc-dVenus*+ and bulk-tissue expression from nine simulations from panel A, each panel showing one simulation with randomly selected genes.

Supplementary Table 1. Gene-by-gene RNA-Seq results. Raw read counts, counts per million reads, log10 fold-change (*Arc-dVenus* over tissue), *p* values, and RefSeq transcript ID for each gene for each sample. The first tab includes all genes analyzed. Subsequent tabs contain subsets of genes that are *Arc-dVenus*-enriched: those that are also activity-regulated (tab 2), visual stimulus-regulated (tab 3), known marker genes for excitatory neurons (tab 4), or genes enriched in *Fos*+ hippocampal neurons (tab 5). *P* values are from edgeR[44].
